# Autism Early Intervention – Progress, Steps Backward, and the Reconciliation of Conflicting Narratives

**DOI:** 10.1007/s11920-024-01552-x

**Published:** 2024-10-25

**Authors:** Giacomo Vivanti

**Affiliations:** https://ror.org/04bdffz58 grid.166341.7 0000 0001 2181 3113 A.J. Drexel Autism Institute, Drexel University, 3020 Market Street, Suite 560, Philadelphia, PA 19104 USA

**Keywords:** Autism, Early intervention, Evidence-based, Profound autism, Neurodiversity

## Abstract

**Purpose of Review:**

We review recent research on interventions, services and supports for children on the autism spectrum, examining both advancements and setbacks.

**Recent Findings:**

Progress has included an increase in quantity and rigor of intervention science, as well as a broadening of disciplines and perspectives engaged in the examination of early interventions, including their effectiveness, social validity and the contextual determinants of implementation outcomes. Setbacks have included the decrease in research involving children on the autism spectrum who have co-occurring profound intellectual disability, minimal or no spoken language, and who require constant assistance with daily living activities. This trend is alarming because it contributes to the marginalization and unmet needs of children who need intervention the most. Additionally, access to early intervention services is unequal and complicated by the misalignment of policy with the evolving evidence base in the field.

**Summary:**

The recent growth in the depth and breadth of knowledge related to autism early intervention means that policies, practices, advocacy efforts and research directions can be grounded on a more comprehensive evidence base and societal appraisal of autism. Nevertheless, these indisputable markers of success co-exist with conflicting narratives that hinder the establishment of a cohesive agenda to tackle inequities experienced by marginalized subgroups. Reconciliation of conflicting narratives requires a nuanced and compassionate appraisal of sources of tensions and heterogeneity of needs within the autism spectrum.

## Introduction – Recent Progress in Autism Early Intervention

Historically, advances in the field of intervention have lagged behind other areas of autism research [1]. In the past decade, research on interventions, services and supports for children on the autism spectrum has seen a substantial growth across several dimensions, including the number of interventions and practices being tested and the methodological standards of intervention trials [2]. Additionally, the scope of early autism intervention research has broadened beyond the examination of efficacy, including a focus on how intervention outcomes are modulated by individual factors [3, 4], as well as contextual factors such as implementation fidelity [5], social determinants [6], and service providers’ organizational climate [7]. Relatedly, the field is witnessing an increased focus on the processes through which interventions exert their effects, including the articulation of mechanistic theories [8], and empirical investigation that tests hypothesized mechanisms of change for different interventions [9]. Advances in these areas are providing actionable insight on ‘what works for whom and why’, thus paving the way for the proactive assignment of children to interventions that are more likely to be beneficial for them [3, 9]. Additionally, this line of research can facilitate the flexible adoption of interventions in the community by characterizing their ‘active ingredients’ as well as the elements that might be ‘safely’ removed, adapted, optimized, and customized without diluting their effectiveness [10, 11].

Relatedly, there is an increasing focus on the implementation and evaluation of autism early interventions within community services, including early childhood education settings [12] and early intervention systems [13, 14], as well as research examining *why* specific practices are preferred by specific providers and used for specific service recipients in the community [15–17]. Understanding how the adoption and outcomes of early interventions are shaped by the interplay between characteristics of children, features of the intervention, and features of the implementation context is critical to the development of personalized intervention algorithms and stepped care approaches, contributing to a more nuanced and less dogmatic appraisal and deployment of interventions.

Further, a growing number of studies have examined the acceptability of early intervention practices and targets for children on the autism spectrum, their families, and the intervention providers across cultural and geographical contexts [18, 19] and through the lens of an increasing range of disciplines (e.g., implementation science, applied behavior analysis, disability studies [20]) and perspectives (e.g., the neurodiversity approaches [21]). For example, a small but growing literature has focused on evaluating alignment between interventions based on applied behavior analysis (ABA) and concepts that have emerged within social models of disability and neurodiversity-affirming forums [22–24]. These ‘bridging’ efforts based on community-based participatory research and stakeholders’ engagement have shown a nuanced picture of acceptability in relation to different behavioral practices and goals, rather than incompatibility *tout-court* [25–27]. This points to the notion that the goal of honoring and valuing identities and ways of being that do not conform to neurotypical standards is not antithetic to addressing manifestations of autism that do pose disabling barriers to learning and wellbeing, including pathognomonic communication challenges [26].

Overall, the recent growth in the depth and breadth of knowledge related to autism early intervention means that policies, practices, advocacy efforts and research directions can be grounded on a more comprehensive evidence base and societal appraisal of autism. Nevertheless, these indisputable markers of success co-exist with steps backward and setbacks.

## Steps Backward – The Increasing Marginalization of Profound Autism

Needs-based approaches to services and supports are centered on the notion that research, practice and policy should allocate increased attention to those with the most profound needs [28–30]. However, in the autism intervention field the trend is in the opposite direction, with an *escalating* exclusion from research of children with profound intellectual disability and minimal spoken language who require constant assistance with daily living activities [31–33]– a presentation recently defined as ‘profound autism’ [34]^1^. This trend is alarming because under-representation in research exacerbates marginalization, perpetuates unmet needs, and impedes development of targeted interventions for children who need help the most. Restrictive exclusion criteria in intervention research based on IQ levels and co-occurring conditions (e.g., epilepsy, preterm birth) contribute substantially to this phenomenon [37, 38]. Additionally, under-representation of profound autism extends beyond research, as media portrayals privilege manifestations of autism that are less dissimilar to neurotypical standards of ability [39].

However, the trend towards decreasing representation in research for those with more intensive intervention needs might also reflect a societal and scientific climate that makes it increasingly difficult to advocate for this marginalized subgroup. One aspect of this climate is the perception that research focused on the intervention needs of those with profound autism is misaligned with a neurodiversity-affirming intervention agenda, i.e., the promotion of intervention practices that do not pathologize autism, capitalize on strengths and preferences, and embrace diversity and affirming care. As discussed later in the article, this perception is arguably misguided, but it affects advances in the field by discouraging targeted advocacy and research efforts for those with more intense service needs.

Speaking from personal experience for a moment, this reality became clear to me during a workshop on neurodiversity affirming research in which I mentioned the unmet needs of my two twin brothers – two adults on the autism spectrum, who, along with above-average personal qualities such as candidness and resilience, have profound disabilities in the areas of receptive and expressive communication, cognition and adaptive behavior. Despite their need for 24/7 care to ensure their safety, hygiene and wellbeing, they are not receiving any services or supports other than assistance from immediate family, after the few targeted programs available in their community proved to be unable or unwilling to address their complex needs. My aim in sharing this painful reality during the meeting (which was expressed avoiding the term ‘profound autism’ to minimize the risk of being attacked over terminology) was to illustrate the need for counteracting the under-representation of those like my brothers in intervention research within a neurodiversity affirming framework; my brothers’ disability is not inherently at odds with living a happy life, but the scale of their needs largely exceeds the services and supports available to them – thus, intervention research centered on those like them is critical for advancing practices and policies to address their needs.

Notably, the response from the workshop participants focused not so much on deploring the lack of services available to my brothers, but on questioning the reality of their impairments. This included the suggestion that my brothers *chose* not to use spoken language because they were misunderstood or abused from neurotypical family members, that their “apparent” communication and cognitive disabilities resulted from the double-empathy problem, whereby other autistics would be perfectly equipped, unlike their family members, to understand and address their needs, and finally, if their intellectual or language challenges were real, then their problem was not “the autism”, but unrelated cognitive and language impairments. This episode, offered here as an example for what has become a common experience [40, 41], deserves a closer examination because it gives insight on the challenges of redressing the escalating trend towards unequal representation of profound autism in research. I will gloss over the resurgence of the 1950s-era ‘inadequate parenting’ narrative, as well as the dramatic irony in the notion that other autistics would be knowledgeable in addressing my brothers’ needs (the only services they have received in their lives involved an almost exclusive contact with other autistics, and that did not change their need for 24/7 care to ensure their safety, or the fact that in practical terms no one other than immediate family is there to support their needs).

I will focus instead on a more insidious element, which is the ableism implicit in the resistance to accept either *the reality* or *the value* of those whose level of ability in the cognitive and/or receptive communication domain differs from standards of typical performance or desirable narratives of success. Moving from personal experience to scholarship, this issue is exemplified by a passage from ‘Neurotribes’ [42]. In a chapter devoted to autism during the Nazi era, the author recounts the medical murder of Alfred, a boy who was ‘euthanized’ in his clinical facility because he failed to develop speech. Revealingly, this case illustration of the Nazi-era atrocities is accompanied by the qualification that Alfred was non-speaking but “was highly intelligent and understood everything” (page 135). The unnecessary emphasis on Alfred’s unimpaired cognitive and receptive language is inexplicable, unless it is implied that his murder would qualify as a lesser atrocity should have his intelligence or language understanding been impaired. Similar accounts that either deny or devalue the status of those on the autism spectrum who do not meet the ‘desirable’ cognitive and communication neurotypical standards are pervasive in autism discourse [43], shaping a climate in which those like my brothers are misguidedly perceived as an unwelcomed poor fit to neurodiversity affirming narratives. This, in turn, discourages a focus on their needs, which perpetuates and exacerbates their disability.

Changing this climate requires an intimidation-free and collaborative effort, including setting aside ambiguities on whether the escalating under-representation of children with profound impairments requiring 24/7 assistance with daily living activities is indeed a concern for all the autism community, or only for special interest groups who are directly experiencing this reality. Acknowledging and prioritizing the needs of those like my brothers does not mean taking airtime away from celebrating autistic strengths or from addressing the needs of those on the autism spectrum who experience different priorities (for example, the societal pressure to conform for those who, unlike my brothers, can decide to mask their condition). Rather, a focus on profound autism affirms unequivocal commitment to a neurodiversity affirming agenda – at least within a compassionate and inclusive conceptualization of neurodiversity that values the wellbeing of each person on the autism spectrum, regardless of their perceived fit with hashtag-ready success stories of “normal” or superior abilities.

Importantly, the impairments of children in this vulnerable subgroup do co-exist with strengths, which should be celebrated *especially* for those with severe intellectual and communication disabilities; relatedly, interventions should capitalize on those strengths, value children’s preferences and agency, and acknowledge the disabling impact of societal norms and structures on their lives. However, even in a hypothetically just and enlightened society, the disabling impairments of those like my brothers would not cease to exist or to cause the need for 24/7 support. These children will be increasingly excluded from benefitting from research if their reality continues to fade from the radar of intervention research and societal discourse. To counteract this trend, targeted research and advocacy is needed that acknowledges the scale of their needs and promotes a scientifically rigorous and compassionate agenda centered around their wellbeing.

## One Area of Stagnation: Alignment of Evidence and Policy

One area of stagnation in the field concerns the alignment of service provision policy with the evolving evidence on early intervention. This issue is exemplified by mandate legislation that, in many US states, directly ties funding eligibility to ABA-based interventions [44]. Although ABA-based strategies have been shown to be beneficial for many children [45], this policy is arguably problematic for two reasons. First, there is ambiguity on which interventions should be subsumed (and consequently covered) under the umbrella of ABA [46, 47], and second, empirical support exists for early interventions that are not exclusively based on ABA principles [2, 48, 49].

The first issue pertains to the lack of a shared conceptual taxonomy of early interventions for autism [50]. This is exemplified by the fact that recent systematic reviews and meta-analyses in this area use each a different approach to classify interventions, including variable operationalization criteria for the ABA umbrella [2, 49, 51–55]. For example, in the recent meta-analysis by Wergeland et al. [55], a trial testing the effectiveness of the Joint Attention Mediated Learning intervention [56] was included as supporting evidence for “Behavioral Interventions”, while in the meta-analysis by Sandbank et al. [49] the same trial was used to evaluate the evidence for “Developmental Interventions”, a category defined as mutually exclusive with “Behavioral Interventions”. Other reviews have included the same intervention as an exemplar of other categories yet [57].

Another example concerns the Early Start Denver Model (ESDM [58]), which is included by some reviews as an exemplar of ABA practice [59], whereas in other studies it is placed outside of the ABA umbrella [60]. This ambiguity means that the ESDM can be characterized as qualifying for funding purposes depending on the sources that decision-makers rely on to evaluate its alignment with ABA. This results in (a) families being arbitrarily granted or denied coverage for ESDM services (the same is true for other evidence-based practices), and (b) service providers hesitating in adopting ESDM or other practices regardless of their evidence, because uncertainty on their inclusion under the ABA umbrella results in unpredictable determinations of funding eligibility [47]. As an example, a provider inquiring about insurance coverage for ESDM recently shared that she was not “*able to find out whether there is a consensus in the field about the status of ESDM. The Association of Professional Behavior Analysts informed me that the organization has not taken an official position on the ESDM*”.

To overcome reliance on idiosyncratic local determinations, the early intervention field could establish consensus on a parsimonious set of intervention categories and operationalizations that include the degree of conceptual alignment to the principles of ABA. Along these lines, a distinction can be drawn between “Early Intensive Behavioral Intervention” (EIBI), whose conceptual apparatus and operational procedures are *exclusively* informed by ABA; (b) Naturalistic Developmental Behavioral Interventions (NDBIs), which includes both ABA concepts and strategies and empirical knowledge derived from non-ABA disciplines, including developmental psychology and infant mental health [61, 62], and (c) Developmental Interventions, which are not explicitly informed by ABA literature (e.g [63, 64]. Against this background, clarity is needed on whether coverage for ABA is interpreted as coverage for EIBI only or other categories as well.

Relatedly, interventions in the “Developmental Interventions” category, such as PACT [63], also have shown evidence for effectiveness [65], pointing to the importance of determining intervention coverage eligibility based on an agnostic appraisal of each intervention’s evidence-based status, rather than alignment with ABA principles per se. One obstacle to this modernization agenda is that the concepts of “evidence-based” and “ABA practice” are often conflated [66, 67]. This conflation seems a vestigial reflection of the historical contrast between behaviorism’s commitment to scientific rigor and the rejection of quantitative science expressed by the psychoanalytical establishment that dominated the field when behavioral interventions for autism were first pioneered [68]. In some sectors of the scientific community, these historical circumstances contributed to shape a long-lasting rhetoric equating endorsement of any non-ABA practice with a rejection of science [66, 69], posing an obstacle towards the goal of critically appraising new empirical knowledge [70], legitimizing and reconciling different perspectives [47], and advocating for the alignment of policies with evolving empirical research [67].

The lack of shared taxonomies for classifying interventions, coupled with the lack of shared parameters for evaluating their effectiveness [71], and conflicting narratives surrounding ABA [68] means that end-users and policy makers are exposed to different “versions of reality” on the alignment of interventions with funding mandates (as well as their evidence-based status and alignment with stakeholders’ priorities), contributing to difficulties in translating evolving knowledge into practice and policy. As discussed in the following paragraph, a reconciliation agenda is key to acknowledge and address this and the other conflicting narratives that complicate progress in the autism intervention field.

## Conclusions - Embracing Nuance to Advance a Reconciliation Agenda

The past decade has witnessed unprecedented vitality in the study of autism intervention, reflecting the expanding evidence base and landscape of disciplines and perspectives engaged with autism. However, coalescing data and insights from different perspectives into a cohesive framework for action is becoming an increasingly complex endeavor. Challenges include distilling scientific ‘truth’ from multiple and at times conflicting sources of knowledge, extracting usable information that is relevant across the range of phenomena and experiences related to autism, and identifying priorities and gaps in the literature that are ‘buried’ within the noise of conflicting narratives and the exponential increase of information related to autism.

This task is complicated by a climate of increasingly heightened sensitivities, partly due to the emotionally-charged yet conceptually fuzzy nature of the constructs causing contention in the field, such as neurodiversity-affirming practices, ABA-based interventions, and evidence-base practice [46]. The antagonistic atmosphere permeating scholarship on these topics influences, and is influenced by, the tone of online forums and social media discussions, where combative statements that foster “us versus them” narratives are rewarded over critical reflections that acknowledge complexity [71, 72]. In contrast, what the field of autism intervention needs the most is to embrace nuance.

As the scientific investigation and societal discourse on autism evolve and broaden, it is important to recognize that any appraisal of the state of knowledge in the field, just like any specific lived experience, will only partially capture the reality of autism. Since both the phenomena subsumed under the ‘autism intervention’ construct and the lenses through which these phenomena are approached have multiplied, cross-disciplinary and cross-stakeholder efforts are needed to counteract the calcification of attitudes and beliefs in the field, including assumptions on the evidence, categorization and acceptability (or lack of thereof) for specific interventions [70]. Such collaborative efforts can promote a more context-conscious and nuanced appraisal of alignment and misalignment between policy, practices, scientific evidence, as well as stakeholders’ views on acceptability and agendas, and encourage a more agnostic ‘dissection’ of specific elements of contention and their relevance for specific children and contexts.

Relatedly, a collaborative commitment is needed to unambiguously acknowledge and address the barriers that prevent participation in intervention research for those needing intervention the most [73]. These include not only children with more profound cognitive and communication impairments, but also other marginalized subgroups, such as economically disadvantaged individuals, as well as racial and ethnic minoritized groups. Efforts to prevent or mitigate these barriers require rigorous research informed by different disciplinary perspectives and by the experiences of autistic people across the spectrum of manifestations of autism, their families and the individuals and agencies interfacing with them (service providers, educators in the school system, healthcare practitioners). These efforts offer prospects for wide-ranging progress in the field, if grounded on research methods, priorities and metrics that cut across different agendas and respectfully acknowledge the multitude of phenomena, constructs and experiences related to autism.

## Data Availability

No datasets were generated or analysed during the current study.

## Footnotes

1 Although in its original formulation the term ‘profound autism’ was intended to describe children older than 8, recent research has shown that cognitive and adaptive functioning in toddlerhood are predictive of later outcomes [35, 36], leading to extending the concept to younger ages and pointing to the impact of excluding children with profound needs in cognitive and adaptive domains from early intervention trials.
